# Influencing Factors of Students Aged 10–20 Non-participating in Home Physical Exercise During the COVID-19 Isolation Policy Period: A Cross-Sectional Study From China

**DOI:** 10.3389/fpubh.2022.787857

**Published:** 2022-06-15

**Authors:** Lin Luo, Xiaojin Zeng, Yan Wu, Fei An, Jiahong Huang, Hao Yang, Quanning Jiang, Qiang Ou, Jianjun Du, Naiqing Song

**Affiliations:** ^1^College of Physical Education, Guizhou Normal University, Guiyang, China; ^2^Basic Education Research Center, Southwest University, Chongqing, China; ^3^East China Normal University-Xuhui Postdoctoral Workstation, Shanghai, China; ^4^Zhongxu School Affiliated to East China Normal University, Chongqing, China

**Keywords:** COVID-19, isolation policy, student, family physical exercise, exercise habits

## Abstract

**Background:**

A number of public health measures are required during the COVID-19 pandemic. To stop the spread of COVID-19, the Chinese government has adopted isolation policies, including closing non-essential businesses, public transportation and schools, moving students' face-to-face learning to online, and recommending the cancellation of all non-essential activities and outdoor activities. However, while this isolation strategy has reduced human-to-human transmission of COVID-19, it has led to dramatic changes in students' daily lives and learning styles, including reduced physical activity and increased sedentary time. Considering the potentially harmful effects of physical inactivity, this study hoped to explore the incidence and influencing factors of non-participation in home physical exercise among Chinese students aged 10–20 during the implementation of the COVID-19 isolation policy.

**Methods:**

Through an online questionnaire platform, this study created an open-ended questionnaire (from March 1, 2020 to March 10, 2020) and distributed it to students in areas where isolation policies were enforced. The questionnaire was initially distributed by 10 recruited volunteers, and then the questionnaire was voluntarily forwarded and shared by the subjects or others, in a “snowball” way, to expand distribution. Finally, the survey data of 4,532 Chinese students aged 10–20 were collected. The incidence of respondents non-participating in home physical activity was determined using univariate analysis. Using odds ratios and 95% confidence intervals of a multivariate binary logistic regression model, factors influencing non-participation in home physical exercise were estimated.

**Results:**

Among the sample students, the incidence rate of non-participating in home physical exercise was 25.86% (24.06–27.15%). Exercise intentions, exercise habits, self-assessed health, beliefs in physical health, family exercise, family exercise recommendations, home exercise conditions, school exercise guidance, and health education programs had a negative impact on students non-participating in home physical exercise. Academic performance and electronic product use had a positive effect on non-participating in home physical exercise.

**Conclusions:**

A variety of forward leaning factors, enabling factors and demand factors have affected the occurrence of students” non-participating in home physical exercise. Future health isolation policies should take into account these influencing factors to reduce the occurrence of students” non-participating in home physical exercise and to promote students' independent participation in physical exercise.

## Introduction

The coronavirus (COVID-19) outbreak that began in December 2019 has become a global threat (1). In order to control the infection rate, the governments of China (2), Italy (3), Spain (4) and other countries have adopted isolation policies. Isolation has been the main control strategy for unforeseen epidemic outbreaks (4). In the face of infectious diseases, the general population may not choose to seek medical help because of an inability to recognize symptoms of disease or be aware of the risk of infection (5, 6). This may increase the risk of infection in people who are susceptible to the disease. Previous studies on other infectious respiratory disease pandemics have shown that closing some public places and schools and requiring people to stay at home as much as possible can effectively reduce infection rates (7, 8). In the early stages of the COVID-19 pandemic, the Chinese government launched an unprecedented emergency plan that included closing unnecessary businesses, public transportation and schools as well as transforming students' face to face learning into online learning. At the same time, the public is advised to cancel all non-essential activities and outdoor activities and isolate themselves at home as much as possible to reduce the rapid spread of infection and disease. Initially, this quarantine policy was only enforced in the Wuhan region of China, where the COVID-19 epidemic was most severe. With the rapid spread of COVID-19 across the country, the vast majority of China has begun to implement this quarantine policy. Although this isolation strategy reduces the chance of human-to-human transmission of COVID-19, it increases physical inactivity and sedentary behavior (9–12), and increases the occurrence of adverse psychological conditions, such as insecurity and fear can cause anxiety and stress (13, 14), thoughts of death, feelings of helplessness and abandonment (12). People spend more screen time during isolation (4), and sleep problems and circadian rhythm disturbances increase (4, 15).

In fact, it is important to maintain a certain level of physical exercise during isolation. On the one hand, physical exercise can modulate immune responses in people, especially those who are sedentary and inactive (16, 17). Numerous cross-sectional and longitudinal studies have demonstrated that regular physical exercise has multiple anti-inflammatory effects that may prevent all-cause mortality (18). A mouse experiment showed that moderate endurance exercise (30 min/day) could protect mice from influenza death (19). Older adults may have improved influenza vaccination responses after 10 months of moderate-intensity endurance training (20). At the same time, regular physical exercise has a wide range of mental health benefits, ranging from promoting mental health by improving emotional state (21), to reducing anxiety levels and perceived stress (22, 23). In addition, physical exercise can improve people's sleep status through a variety of psychophysiological pathways (15). In contrast, isolation is known to negatively impact individual immunity, for example by elevating glucocorticoids such as cortisol (24) and suppressing T-cell action (25). And T cells are critical effector lymphocytes that protect vulnerable areas such as the upper airway and lungs (26). Therefore, it is necessary to increase physical exercise during the isolation period.

However, despite the government's advice through the public media to maintain a certain level of physical exercise during the isolation policy of the COVID-19 pandemic, such social distancing measures have greatly reduced opportunities for physical activity in the general population and the incidence of physical inactivity very high (27, 28). During the COVID-19 pandemic, the majority of Chinese stayed at home 20–24 h a day (84.7%) (13). Zhang et al. found that PA in children and adolescents was at very low levels during the COVID-19 epidemic (29). However, less-reported studies exist on factors that may hinder home physical activity during isolation policies. Understanding the characteristics and related factors of people who did not engage in home physical exercise during the isolation policy period may be more conducive to formulating future infectious disease quarantine home health guidelines and policies. Considering the potentially harmful effects of physical inactivity, this study hopes to explore the incidence and influencing factors of non-participation in home physical exercise among Chinese students during the implementation of the COVID-19 isolation policy. These data may contribute to the future development of targeted empirical evidence to strengthen public health policy and guidance on pandemic containment.

**Figure 1:**
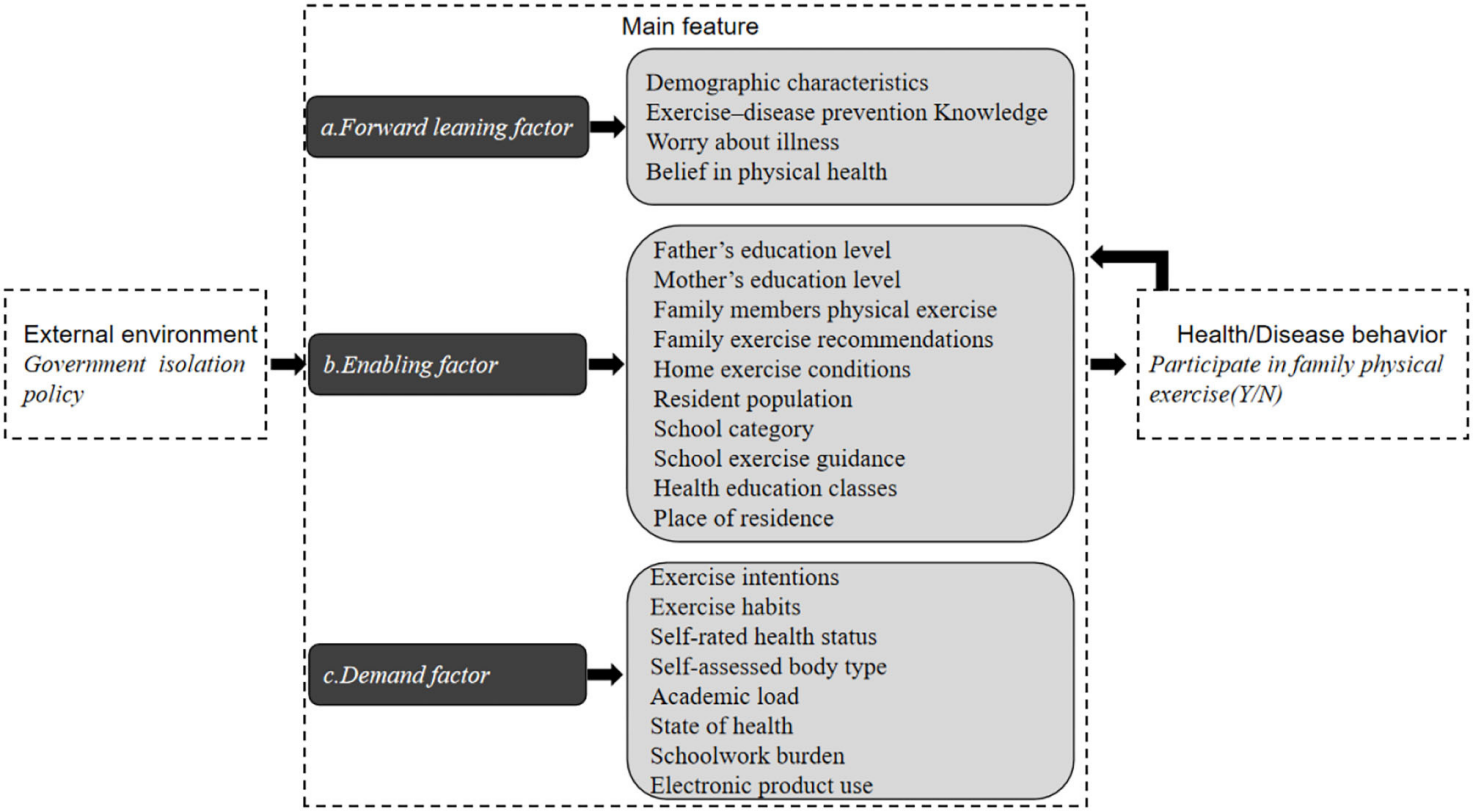
Theoretical framework model of this study.

In order to achieve the research objectives, this study mainly refers to the Anderson health/disease behavior model (30), and designed the questionnaire for this study. The Anderson health/disease behavior model has three components, including external environment, main feature, and health/disease behavior. The external environment and main feature determine health/disease behavior, which in turn affects main feature. The main features include forward leaning factors, enabling factors and demand factors. As shown in Figure 1, the theoretical framework analyzed in this study consists of three parts: the first is the external environment, which mainly refers to the important environmental variables that affect students who do not participate in home physical exercise. The second is the main feature, including forward leaning factors, enabling factors, and demand factors. The third is health/illness behavior, that is, whether the students participate in home physical exercise behaviors or not. As this study was planned, most regions in China had adopted COVID-19 isolation policies. Therefore, the external environment is mainly the isolation policy environment adopted by the state and governments at all levels. This study hypothesizes that forward leaning factors, enabling factors, and demand factors will all have a significant impact on students non-participating in home physical exercise.

## Materials and Methods

### Procedures

The subject research team evaluated the existing literature on student physical activity/inactivity and finally developed the initial questionnaire for this study. To determine the validity of the questionnaire, a panel of experts from four fields of preventive medicine, health science, epidemiology, and physical education was invited to help with the validity assessment (six people in total, four people have senior titles, two people have deputy senior titles). In order to keep the experts' understanding of the evaluation indicators (relevance, clarity, comprehensiveness) of the content validity of the questionnaire consistent, the research team explained the definition of each observation in the questionnaire to the expert panelists. After collecting expert opinions, the subject research team revised the descriptions of some issues/deleted some issues based on the feedback. The research team then invited another 10 student volunteers to help evaluate how the questionnaires were described. Volunteers independently completed questionnaires and provided suggestions for difficulties understanding the descriptions of the questions or answers. In this study, questionnaires were used to collect information on subjects' main characteristics and physical activity. The main feature includes forward leaning factor (exercise-disease prevention knowledge, worry about illness and belief in physical health), enabling factor (father's education level, mother's education level, family members physical exercise, family exercise recommendations, home exercise conditions, resident population, school category, school exercise guidance, health education classes and place of residence), demand factor (exercise intentions, exercise habits, self-rated health status, self-assessed body type, academic load, state of health, schoolwork burden and electronic product use). The survey was conducted from March 1, 2020 to March 10, 2020. This period coincides with the COVID-19 pandemic in China (more than 30,000 confirmed cases and isolation policies in nearly 30 provinces across the country). This study used the Mike CRM website (www.mikecrm.com) to deliver the questionnaire. The Mike CRM website is a platform equivalent to Amazon Mechanical Turk. Participants voluntarily fill out questionnaires via their mobile phones or computers. Considering the fact that Chinese students use mobile phones or computers, this questionnaire only collects data on students in grades K7–12 in areas where the isolation policy is implemented. The KMO of the questionnaire was 8,021, the Bartlett test *p* < 0.05, and the Cronbach alpha coefficient was 0.830.

### Data Collection

#### Demographic Characteristics

The study collected participants' genders, ages (years) and grades (K7–12).

#### Non-participating in Home Physical Exercise

Home physical exercise was constructed as a binary variable with two outcomes according to the average number of days of exercise per week in the past month: Non-participating in home physical exercise (0 days) or participating in home physical exercise (1 day or more). Home physical exercise is defined as including aerobics, yoga, functional training, power cycling, skipping and other physical activities that can be carried out in the home.

#### Exercise–Disease Prevention Knowledge

In the survey questionnaire, the students were directly asked to agree with the statement “moderate physical exercise is beneficial to the body's ability to resist diseases (31).” According to their answers, values ranging from 1 to 5 were assigned in order to indicate agreement with knowledge about exercise and disease prevention.

#### Exercise Intentions

The exercise intention scale (32) was used to measure the exercise intentions of middle school students. We used the following rules to construct exercise intentions as three categorical variables: A score below *P*_25_ was considered a low level, a score of *P*_25_~*P*_75_ was medium and a score above was high (see Supplementary Materials).

#### Exercise Habits

The exercise habits scale (32) was used to measure the exercise habits of middle school students. We used the following rules to construct exercise intentions as three categorical variables: A score below *P*_25_ was considered a low level, a score of *P*_25~_*P*_75_ was medium and a score above *P*_75_ was high (see Supplementary Materials).

#### Worry About Illness

The study asked, “Are you worried about contracting new coronary pneumonia?” in order to determine the level of concerns among middle school students about contracting COVID-19. 0 means not worried, 1 means worried.

#### Self-Rated Health Status

This study used the question, “How do you feel about your overall health now?” in order to measure the self-rated health status of middle school students (33). Respondents answered according to their actual situation. Scores range from one (very bad) to five (very good).

#### Self-Assessed Body Type

This study directly adopted, “Do you think your body belongs to the following type of body type?” to measure the middle school students' judgments regarding their own body types (34). Scores range from one (thin) to three (obese).

#### Belief in Physical Health

We used the Physical Health Belief Questionnaire (35) to measure the physical health beliefs of middle school students. We used the following rules to construct exercise intentions as three categorical variables: a score below *P*_25_ was considered a low level, a score of *P*_25~_*P*_75_ was medium and a score above *P*_75_ was high (see Supplementary Materials).

#### Father's Education Level

Father's education level is divided into five categories: junior high school and below, high school/secondary vocational/higher vocational, junior college, undergraduate and postgraduate and above (36).

#### Mother's Education Level

Mother's education level is divided into five categories: junior high school and below, high school/secondary vocational/higher vocational, junior college, undergraduate and postgraduate and above (36).

#### Family Members Physical Exercise

This study directly adopted, “During the quarantine policy, are there any family members in your family exercising at home?” asking about the physical exercise behaviors of the family members of middle school students. Scores range from 0 (no) to 1 (yes).

#### Family Exercise Recommendations

This study directly adopted, “Did your family advise you to take physical exercises during the isolation policy period?” to determine family advice regarding exercise for middle school students. Scores range from 0 (no) to 1 (yes).

#### Home Exercise Conditions

This study directly adopted “Do you have the conditions for home exercise in your home?” to ask about the home exercise environments of middle school students. Scores range from 0 (no) to 1 (yes).

#### Resident Population

This study directly adopted “How many people currently live in your family?” (37) to ask about the number of family members living with middle school students. Scores range from 1 (0 person) to 7 (6 persons and above).

#### School Category

School category is divided into non-public and public.

#### School Exercise Guidance

This study directly adopted “Did the school send you physical exercise guidance information during the epidemic period?” to ask about school physical exercise guidance for middle school students. Scores range from 0 (no) to 1 (yes).

#### Health Education Classes

This study directly adopted “Did the school have any health education courses during the epidemic period?” to ask about health education courses for middle school students. Scores range from 0 (no) to 1 (yes).

#### Schoolwork Burden

During the survey period, online teaching had already begun in each school. This study operationalized the burden of schoolwork into the daily learning time (hours) reported by the survey respondents (38). Scores range from 1 (within 0.5 h) to 7 (more than 5 h).

#### Place of Residence

The place of residence is divided into three categories: rural, township and urban.

#### Electronic Product Use

To investigate the impact of “mediatization of daily life,” this study takes the operationalization of the use of electronic products as an orderly categorical variable measured as the daily usage time of electronic products that was reported through feedback from the survey respondents (39). Scores range from 1 (within 0.5 h) to 7 (more than 5 h).

### Data Analysis

The first part is a descriptive analysis of the observed variables in this study using the Kolmogorov–Smirnov test for normality. The description of normal measurement data uses Mean ± SD. The counting data is reported as frequencies and percentages (%). An independent sample *t-*test and a chi-square test were used to compare the differences in family physical exercise behavior between groups. The correlation between each observation index and the family physical exercise behavior of middle school students was tested using a multivariate binary logistic regression model based on an odds ratio (OR) and a 95% confidence interval (CI). All values are two-tailed values, and the significance level is 0.05.

## Results

### Characteristics of Participants

The online questionnaire for this study was viewed 16,890 times, and 4,532 questionnaires that met the research conditions were received with valid feedback. In the sample, there are 2,334 girls and 2,198 boys. There are 1,880 rural students and 2,652 urban students. There are 678 students in K7, 777 in K8, 824 in K9, 893 in K10, 738 in K11, and 622 in K12. The age of the students ranged from 10 to 20 years old, with an average age of 15.63 ± 1.80 years. There are 3,745 students in public schools and 787 students in private schools. Of the 4,532 participants, 1,172 students self-reported not participating in home physical activity. Taking students who participated in home physical exercise as the reference group, the average age of students who did not participate in home physical exercise was older. The two groups of students differed in the frequency percentages of the following observed variables, including gender, grade, exercise-disease prevention knowledge, belief in physical health and health concept, father's education level, mother's education level, family members physical exercise, family exercise recommendations, home exercise conditions, school category, school exercise guidance, health education classes and place of residence, exercise intentions, exercise habits, self-rated health status, self-assessed body type, academic load, state of health, schoolwork burden and electronic product use (Table 1).

**Table 1:** Summary statistics on the characteristics of participants.

**Variables**		**No HPA (*N* = 1,172)**	**HPA (*N* = 3,360)**	***X*** **^2^/t-value**	***p*-value**
**Gender**, ***n*** **(%)**				8.699	**0.003**
	Female	647 (55.20)	1,687 (50.21)		
	Male	525 (44.80)	1,673 (49.79)		
**Age (years), mean (SD)**		15.802 (0.048)	15.564 (0.031)	3.893	**<0.001**
**Grade**, ***n*** **(%)**				51.000	**<0.001**
	K7	114 (9.73)	564 (16.79)		
	K8	203 (17.32)	574 (17.08)		
	K9	198 (16.89)	626 (18.01)		
	K10	288 (24.57)	605 (18.01)		
	K11	195 (16.64)	543 (16.16)		
	K12	174 (14.85)	448 (13.33)		
**Exercise disease prevention knowledge**, ***n*** **(%)**				97.637	**<0.001**
	Totally disagree	9 (0.77)	13 (0.39)		
	Mostly disagree	28 (2.39)	31 (0.92)		
	Uncertain	213 (18.17)	333 (9.91)		
	Mostly agree	559 (47.70)	1.543 (45.92)		
	Agree completely	363 (30.97)	1,440 (42.86)		
**Exercise intention**, ***n*** **(%)**				430.326	**<0.001**
	Low	554 (42.27)	623 (18.54)		
	Medium	530 (45.22)	1,878 (55.89)		
	High	88 (7.51)	859 (25.57)		
**Exercise habits**, ***n*** **(%)**				537.501	**<0.001**
	Poor	644 (54.95)	687 (20.45)		
	General	479 (40.87)	2,042 (60.77)		
	Better	49 (4.18)	631 (18.78)		
**Worry about illness**, ***n*** **(%)**				0.005	0.943
	No	282 (24.06)	790 (23.51)		
	Yes	890 (75.94)	2,570 (76.49)		
**Self-rated health status**, ***n*** **(%)**				145.689	**<0.001**
	Very bad	15 (1.28)	13 (0.39)		
	Not too good	43 (3.67)	37 (0.39)		
	General	425 (36.26)	763 (22.71)		
	Better	314 (26.79)	1,021 (30.39)		
	Very good	375 (32.00)	1,526 (45.42)		
**Self-assessed body type**, ***n*** **(%)**				39.734	**<0.001**
	Thin	161 (13.74)	400 (11.90)		
	Normal	681 (58.11)	2,281 (67.89)		
	Obese	330 (28.16)	679 (20.21)		
**Belief in physical health**, ***n*** **(%)**				81.384	**<0.001**
	Low	406 (34.64)	794 (23.63)		
	Medium	587 (50.09)	1,700 (50.60)		
	High	179 (15.27)	866 (25.77)		
**Father's education level**, ***n*** **(%)**				10.796	**0.029**
	Junior high school and below	787 (67.15)	2,094 (63.32)		
	High school/secondary Vocational/higher vocational	182 (15.53)	550 (16.37)		
	College	94 (8.02)	346 (10.30)		
	Undergraduate	94 (8.02)	325 (9.67)		
	Postgraduate and above	15 (1.28)	45 (1.34)		
**Mother's education level**, ***n*** **(%)**				19.372	**0.001**
	Junior high school and below	856 (73.04)	2,228 (66.31)		
	High school/secondary Vocational/higher vocational	140 (11.95)	535 (15.92)		
	College	90 (7.68)	287 (8.54)		
	Undergraduate	77 (6.57)	275 (8.18)		
	Postgraduate and above	9 (0.77)	35 (1.04)		
**Family members physical exercise**, ***n*** **(%)**				448.980	**<0.001**
	No	629 (53.67)	703 (20.92)		
	Yes	543 (46.33)	2,657 (79.08)		
**Family exercise recommendations**, ***n*** **(%)**				346.698	**<** **0.001**
	No	433 (39.95)	414 (12.32)		
	Yes	739 (63.05)	2,946 (87.68)		
**Home exercise conditions**, ***n*** **(%)**				449.026	**<0.001**
	No	847 (72.27)	1,225 (36.46)		
	Yes	325 (27.73)	2,135 (63.54)		
**Resident population**, ***n*** **(%)**				12.036	0.061
	0 person	3 (0.26)	3 (0.09)		
	1 person	6 (0.51)	16 (0.48)		
	2 persons	62 (5.29)	137 (4.08)		
	3 persons	243 (20.73)	770 (22.92)		
	4 persons	343 (29.27)	1,079 (32.11)		
	5 persons	286 (24.40)	730 (21.73)		
	6 persons and above	229 (19.54)	625 (18.60)		
**School category**, ***n*** **(%)**				6.524	**0.011**
	Non-public	175 (14.93)	612 (18.21)		
	Public	997 (85.07)	2,748 (81.29)		
**School exercise guidance**, ***n*** **(%)**				226.010	**<0.001**
	No	568 (48.46)	836 (24.88)		
	Yes	604 (51.54)	2,524 (75.12)		
**Health education class**, ***n*** **(%)**				68.458	**<0.001**
	No	178 (15.19)	238 (7.08)		
	Yes	994 (84.81)	3,122 (92.92)		
**Schoolwork burden**, ***n*** **(%)**				25.745	**<0.001**
	Within 0.5 h	79 (6.74)	234 (6.96)		
	0.5–1 h	150 (12.80)	367 (10.92)		
	1.1~2 h	221 (18.86)	582 (17.32)		
	2.1~3 h	61 (5.20)	169 (5.03)		
	3.1~4 h	55 (4.69)	78 (2.32)		
	4.1~5 h	225 (19.20)	708 (21.07)		
	More than 5 h	381 (32.51)	1,222 (36.37)		
**Place of residence**, ***n*** **(%)**				22.757	**<0.001**
	Rural	545 (46.50)	1,335 (39.73)		
	Town	214 (18.26)	581 (17.29)		
	City	413 (35.24)	1,444 (42.98)		
**Electronic product use**, ***n*** **(%)**				90.953	**<0.001**
	Within 0.5 h	138 (11.77)	611 (18.18)		
	0.5–1 h	198 (16.89)	5,911 (17.59)		
	1.1~2 h	187 (15.96)	534 (15.89)		
	2.1~3 h	77 (6.57)	351 (10.45)		
	3.1~4 h	56 (4.78)	225 (6.70)		
	4.1~5 h	161 (13.74)	401 (11.93)		
	More than 5 h	355 (30.29)	647 (19.26)		

*Bold values represent p < 0.05*.

*No HPA, Non-participating in home physical exercise; HPA, participating in home physical exercise*.

#### Incidence of Students Non-participating in Home Physical Exercise

In the sample, the incidence rate of No HPA students was 25.86% (24.06–27.15%). Female students have a higher proportion of No HPA than male students. Students of 18-year-olds have a higher rate of No HPA than students of other ages. K10 grades have a higher proportion of No HPA than other grades. A higher percentage of No HPA students who answered “ completely disagree/mostly disagree,” with exercise disease prevention knowledge. Students with “low” exercise intention had a higher proportion of No HPA. Students with “poor” exercise habits have a higher proportion of No HPA. Students with self-rated health status as “very poor/not very good” had a higher proportion of No HPA. Students with self-assessed body type as “thin/fat” had a higher proportion of No HPA. Students with “low” belief in physical health had a higher proportion of No HPA. The proportion of No HPA is higher for students whose father is “under junior high school education.” Students whose mothers are “junior high school and below” have a higher proportion of No HPA. The proportion of No HPA was higher for students whose family members, physical exercise as “none,” family exercise recommendations as “none” and home exercise conditions as “none.” Public school students have a higher rate of No HPA. The proportion of No HPA was higher for students with “no” in school exercise guidance and “no” in health education class. The proportion of No HPA is higher for students whose schoolwork burden is “3.1~4 h,” the place of residence is “rural,” and the time of electronic product use is “4.1~5 h/more than 5 h” (Table 2). There was no significant difference in the proportion of No HPA among students with different Worry about illness (*x*^2^ = 0.005, *p* = 0.943) and different resident population (*x*^2^ = 12.036, *p* = 0.061) (see Table 2).

**Table 2:** Summary statistics of incidence of No HPA.

**Variables**		**No HPA**	**X^**2**^-value**	***p*-value**
Total, %		25.86 (24.06~27.15)		
**Gender, %**			8.684	0.003
	Female	27.72 (25.94~29.57)		
	Male	23.89 (22.15~25.71)		
**Age (years), %**			48.763	<0.001
	10	0.00		
	11	25.00 (3.35~26.23)		
	12	15.48 (9.20~24.86)		
	13	18.79 (15.67~22.35)		
	14	23.03 (19.95~26.43)		
	15	25.64 (22.89~28.60)		
	16	30.10 (27.23~33.13)		
	17	29.10 (25.92~32.50)		
	18	30.63 (26.58~35.02)		
	19	21.38 (15.69~28.44)		
	20	15.56 (9.43~24.57)		
**Grade, %**			51.000	<0.001
	K7	16.81 (14.18~19.82)		
	K8	26.13 (23.16~29.33)		
	K9	24.03 (21.23~27.07)		
	K10	32.25 (29.26~35.39)		
	K11	26.42 (23.37~29.72)		
	K12	27.97 (24.59~31.63)		
**Exercise disease prevention knowledge, %**			97.637	<0.001
	Totally disagree	40.91(22.83~61.83)		
	Mostly disagree	47.46 (35.14~60.09)		
	Uncertain	39.01 (35.00~43.17)		
	Mostly agree	26.59 (24.75~28.53)		
	Agree completely	20.13 (18.35~22.05)		
**Exercise intention, %**			430.326	<0.001
	Low	47.07 (44.23~49.93)		
	Medium	22.01 (20.40~23.71)		
	High	9.29 (7.60~11.31)		
**Exercise habits, %**			537.501	<0.001
	Poor	48.38 (45.71~51.07)		
	General	19.00 (17.52~20.58)		
	Better	7.21(5.49~9.41)		
**Self-rated health status, %**			145.689	<0.001
	Very bad	53.57 (35.44~70.81)		
	Not too good	53.75 (42.82~64.33)		
	General	35.77 (33.10~38.54)		
	Better	23.52 (21.32~25.87)		
	Very good	19.73 (18.00~21.58)		
**Self-assessed body type, %**			39.734	<0.001
	Thin	28.70 (25.10~32.58)		
	Normal	22.99 (21.51~24.54)		
	Obese	32.71 (29.88~35.66)		
**Belief in physical health, %**			81.384	<0.001
	Low	33.83 (31.21~36.56)		
	Medium	25.67 (23.92~27.50)		
	High	17.13 (14.96~19.54)		
**Father's education level, %**			10.796	0.029
	Junior high school and below	27.32 (25.72~28.97)		
	High school/secondary Vocational/Higher vocational	24.86 (21.86~28.13)		
	College	21.36 (17.78~25.44)		
	Undergraduate	22.43 (18.69~26.68)		
	Postgraduate and above	25.00 (15.67~37.42)		
**Mother's education level, %**			19.372	0.001
	Junior high school and below	27.76 (26.20~29.36)		
	High school/secondary Vocational/higher vocational	20.74 (17.85~23.97)		
	College	23.87 (19.84~28.44)		
	Undergraduate	21.88 (17.86~26.50)		
	Postgraduate and above	20.45 (11.00~34.86)		
**Family members physical exercise, %**			448.980	<0.001
	No	47.22 (44.55~49.91)		
	Yes	16.97 (15.71~18.31)		
**Family exercise recommendations, %**			346.698	<0.001
	No	51.12 (47.75~54.48)		
	Yes	20.05 (18.79~21.38)		
**Home exercise conditions, %**			449.026	<0.001
	No	40.88 (38.78~43.01)		
	Yes	13.21 (11.93~14.61)		
**School category, %**			6.524	0.011
	Non-public	22.24(19.47~25.28)		
	Public	26.62(25.23~28.06)		
**School exercise guidance, %**			226.010	<0.001
	No	40.46 (37.92~43.05)		
	Yes	19.31 (17.96~20.73)		
**Health education class, %**			68.458	<0.001
	No	42.79 (38.11~47.60)		
	Yes	24.15(22.87~25.48)		
**Schoolwork burden, %**			25.745	<0.001
	Within 0.5 h	25.24 (20.73~3,035)		
	0.5–1 h	29.01(25.26~33.08)		
	1.1~2 h	27.52 (24.54~30.72)		
	2.1~3 h	26.52 (21.22~32.60)		
	3.1~4 h	41.35 (33.30~49.90)		
	4.1~5 h	24.12 (21.48~26.97)		
	More than 5 h	23.77 (21.75~25.91)		
**Place of residence, %**			22.757	<0.001
	Rural	28.99 (26.98~31.08)		
	Town	26.92 (23.95~30.11)		
	City	22.24 (20.41~24.19)		
**Electronic product use, %**			90.953	<0.001
	Within 0.5 h	18.42 (15.81~21.36)		
	0.5–1 h	25.10 (22.19~28.24)		
	1.1~2 h	25.94 (22.87~29.26)		
	2.1~3 h	17.99 (14.63~21.92)		
	3.1~4 h	19.93 (15.66~25.01)		
	4.1~5 h	28.65 (25.06~32.53)		
	More than 5 h	35.43(32.53~38.44)		

*No HPA, Non-participating in home physical exercise*.

#### Trends in the Incidence of On-Participating in Home Physical Exercise Among Students of Different Age Groups

**Figure 2:**
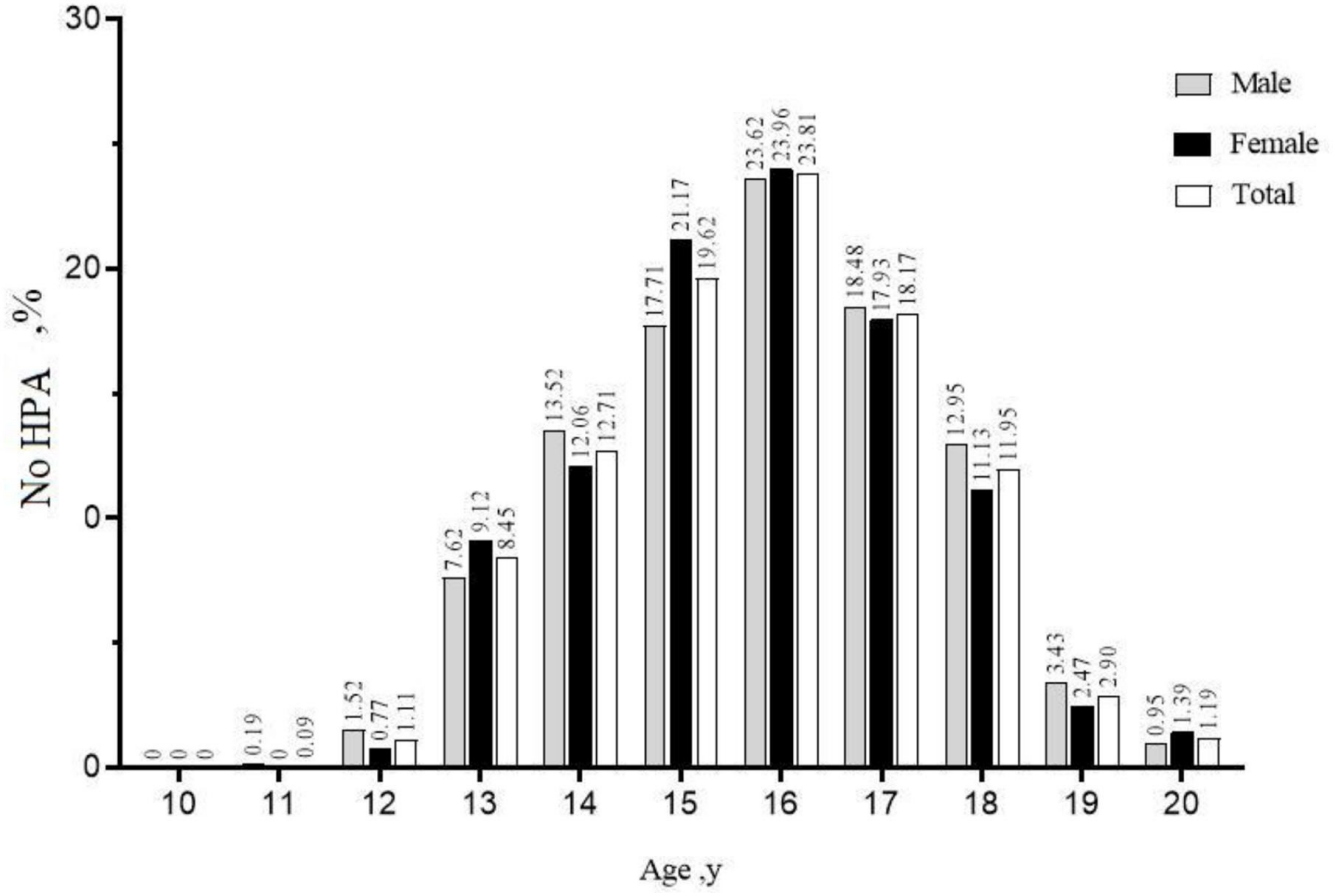
Trends in the incidence of non-participating in home physical exercise in different age subgroups. No HPA, Non-participating in home physical exercise.

In all age groups as a whole, the incidence rate of female middle school students without family physical exercise behavior was 27.72% (25.94~29.57%), which was higher than that of male middle school students at 23.89% (22.15~25.71%) (Table 2). The proportion of middle school students without family physical exercise during the home epidemic-prevention period displays an inverted “U”-shape related to age. The specific manifestation involves a gradual increase from 10 to 16 years old and a gradual decrease from 16 to 20 years old. The trend is the same among male and female middle school students (Figure 2).

#### Influencing Factors of Students Non-participating in Home Physical Exercise

The observation indexes with *P-*value < 0.05 in univariate analysis were used as independent variables, and were included in the multivariate binary logistic regression model for analysis. The model results are shown in Table 3. Regression analysis showed that gender [*OR* = 1.033(95%*CI*:0.879~1.214), *p* = 0.694], age [*OR* = 0.919 (95%*CI*:0.840~1.005), *p* = 0.063], exercise disease prevention knowledge [*OR* = 0.944 (95%*CI*:0.846~1.053), *p* = 0.298], self-assessed body type [*OR* = 1.015 (95%*CI*:0.888~1.159), *p* = 0.829], father's education level [*OR* = 1.007 (95%*CI*:0.890~1.139), *p* = 0.916], mother's education level [*OR* = 1.049 (95%*CI*:0.920~1.196),*p* = 0.477], school category [*OR* = 0.913(95%*CI*:0.729~1.144),*p* = 0.430], schoolwork burden [*OR* = 0.983 (95%*CI*:0.946~1.022), *p* = 0.390], place of residence [*OR* = 1.004 (95%*CI*:0.900~1.120)] and students' home physical exercise behavior were not significantly associated.

**Table 3:** OR and 95% confidence intervals for No HPA.

**Variables**	***OR***	***SE***	***z***	***p*-value**	**(95% CI)**
Grade	1.154	0.060	2.750	0.006	1.042	1.278
Exercise intentions	0.727	0.059	−3.960	<0.001	0.621	0.851
Exercise habits	0.419	0.037	−9.870	<0.001	0.353	0.498
Self-rated health status	0.845	0.038	−3.780	<0.001	0.774	0.922
Belief in physical health	1.155	0.076	−2.200	0.028	1.016	1.314
Family members physical exercise	0.458	0.040	−8.900	<0.001	0.386	0.544
Family exercise recommendations	0.574	0.057	−5.600	<0.001	0.473	0.697
Home exercise conditions	0.365	0.032	−11.490	<0.001	0.307	0.433
School exercise guidance	0.678	0.058	−4.520	<0.001	0.573	0.803
Health education classes	0.694	0.086	−2.940	0.003	0.544	0.886
Electronic product use	1.079	0.020	4.140	<0.001	1.041	1.118

*No HPA, Non-participating in home physical exercise*.

## Discussion

This study provides important, updated evidence on Chinese students' home physical exercise during the isolation policy. Overall, the incidence of non-participating in home physical exercise among students aged 10–20 in our study was 25.86%, which is lower than that found in other Chinese research. And students of different ages have different incidences of not participating in home physical exercise, with the highest proportion of 16-year-old students. A previous study of Chinese adolescents showed that during the COVID-19 pandemic, the prevalence of physically inactive students increased from 21.3 to 65.6% (28). The reason for this difference from our results may be their physical activity assessment tools (IPAQ–SF), which are different than those used in this research. Due to the cancellation of face-to-face physical education classes and the drastic reduction in outdoor activities during the COVID-19 epidemic, most questionnaires on physical activity levels are not suitable for home physical exercise surveys. In addition, our investigation of the home physical exercise behavior of students seems to be more focused on the actual situations of Chinese families during the COVID-19 isolation policy.

Previous studies have found that gender and age are significant factors affecting the physical activity of adolescents during the COVID-19 pandemic (40, 41). However, in this study, although age and gender were not significant factors affecting students without home physical exercise, the results showed that the higher the grade of the student, the greater the risk of non-participating in home physical exercise, which is similar to Kang et al. (41). The research group has conducted surveys on the relationships between exercise intentions, exercise habits and physical health beliefs and the physical activity of students in previous studies. The survey results show that these are significantly positively correlated with the frequency of physical activity of students (42, 43). In the current study also observed that those non-participating in home physical exercise had lower exercise intentions, worse exercise habits and lower levels of health beliefs.

In this study, we observed that the longer the use of electronic products, the higher the risk of a lack of family physical exercise behavior in middle school students. This is consistent with the conclusions of some previous studies (28, 43). Schmidt et al. reported that during the COVID-19 pandemic, adolescents' total amount of leisure screen time increased significantly, and physical activity decreased significantly. The increase in total leisure screen time increased the students' sedentary time, which may have had a negative impact on their physical and mental health, and this deteriorating health may have further reduced their physical activity levels (44). Our research also found that students with poorer self-rated health status had a higher risk of lacking family physical exercise. Self-rated health status is an indicator of health status that can be used indirectly as an individual (45). Vingilis et al. reported that adolescents with better self-rated health status were more physical exercise (46).

This study examines the Anderson health/disease behavior model for the first time to explore the impact of multiple main feature factors on the home physical exercise behavior of Chinese students during the COVID-19 quarantine policy. We found that forward-leaning factors, enabling factors, and demand factors all had a significant impact on students' home physical exercise behavior during the isolation policy period. The study found that students who had no family members physical exercise, no family exercise recommendations, no school exercise guidance, and no Health education classes were more likely to not participate in home physical exercise. At the same time, this study also found that the lack of physical exercise conditions in the home environment also significantly affected students' home physical exercise during the isolation policy. The outbreak of COVID-19 in China coincides with the “Spring Festival,” the most important traditional Chinese festival. During this festival, Chinese families have the habit of getting together. COVID-19 isolation policies have increased the gathering of family members, which has led to a reduction in home physical exercise space, which may also affect students' lack of home physical exercise (47).

This study confirmed factors that may potentially affect students non-participating in home physical exercise during this isolation policy. Forward leaning factor(grade and belief in physical health), enabling factor (family members physical exercise, family exercise recommendations, home exercise conditions, school exercise guidance and health education classes), demand factor (exercise intentions, exercise habits, self-rated health status and electronic product use) and students' home physical exercise behavior were significantly associated. Based on the results of this study, it is suggested that in future health education, it is very important to increase students,” interest in physical exercise, cultivate students,” physical exercise habits, and enhance students,” belief in physical fitness. This may help improve physical activity for students when faced with public health restrictions. At the same time, the future health isolation policy should consider the role of forward leaning factors, enabling factors, and demand factors in promoting students” physical exercise. It is recommended that families and schools be included in the student health education program during the isolation period, which will help students adopt a more active lifestyle in response to the physical and mental adjustment of the isolation policy.

The advantage of this study lies in the large sample size, including students of different genders, grades, and regions (rural, urban, and urban). First, the population of this study is very representative, and the results can be used to support future student quarantine health education strategies and health care strategies for inclusion in quarantine policies due to public health emergencies. This study aims to provide recommendations to help promote physical activity promotion for students in segregated policy settings. Second, the survey data for this study was conducted during the COVID-19 quarantine policy, which occurred during a global pandemic health threat. The results obtained in this unique social model may have certain reference value for the design and implementation of physical activity strategies for global student health isolation policies.

## Conclusion

A variety of forward leaning factors, enabling factors and demand factors have affected the occurrence of students' non-participating in home physical exercise. Future health isolation policies should take into account these influencing factors to reduce the occurrence of students' non-participating in home physical exercise and to promote students' independent participation in physical exercise.

## Limitations

This study also has some research limitations. First, the use of an online survey method, which will miss the collection of data on respondents, who cannot use online questionnaires. Secondly, the dissemination of the questionnaire is carried out in a “snowball” method, which will inevitably lead to skewed distribution of some indicators of the sample, such as the uneven age distribution of the respondents, which may partially affect the stability of the research conclusions. Third, the survey time is relatively short, and the investigators use the recall method to report themselves, which will inevitably lead to memory bias. However, from the research results, it is possible to peek into the important influencing factors of students' family physical exercise behavior under the COVID-19 isolation policy environment, which is of reference value for future research.

## Data Availability Statement

The raw data supporting the conclusions of this article will be made available by the authors, without undue reservation.

## Ethics Statement

The studies involving human participants were reviewed and approved by the Ethics Committee of the Sports College of Guizhou Normal University (No. 20200227). Written informed consent to participate in this study was provided by the participants' legal guardian/next of kin.

## Author Contributions

During the research process, LL had full access to all research data and also analyzed the data and wrote the manuscript. NS participated in the research design. JH, HY, XZ, FA, QO, QJ, and YW collected the data. JD participated in the revision of the paper. All authors have read and approved this version of the article.

## Conflict of Interest

The authors declare that the research was conducted in the absence of any commercial or financial relationships that could be construed as a potential conflict of interest.

## Publisher's Note

All claims expressed in this article are solely those of the authors and do not necessarily represent those of their affiliated organizations, or those of the publisher, the editors and the reviewers. Any product that may be evaluated in this article, or claim that may be made by its manufacturer, is not guaranteed or endorsed by the publisher.
