# Supplementary material for: Influencing Factors of Students Aged 10–20 Non-participating in Home Physical Exercise During the COVID-19 Isolation Policy Period: A Cross-Sectional Study From China
Source: Front Public Health. 2022 Jun 15;10:787857. doi: 10.3389/fpubh.2022.787857 (PMC9240655; doi:10.3389/fpubh.2022.787857)
Supplement: Supplementary file 1 [file Table_1.DOC]

**Supplementary file 1：**

Please read each item carefully to understand its meaning, and then choose the answer that best suits you according to your actual situation, and tick the corresponding number with "√".

(1.Totally inconsistent;2.Not very consistent;3.Some match;4.More in line with;5.Completely suitable )

| **The exercise intention scale** | | | | | |
| --- | --- | --- | --- | --- | --- |
| 1.I am willing to spend my money on exercise | 1□ | 2□ | 3□ | 4£ | 5£ |
| 2.I will persuade people around to exercise with me | 1□ | 2□ | 3□ | 4□ | 5□ |
| 3.I always devote myself to exercise | 1□ | 2□ | 3□ | 4□ | 5□ |
| 4. No matter how many difficulties I encounter, I will keep exercising | 1□ | 2□ | 3□ | 4□ | 5□ |
| 5. I am very satisfied with my continued exercise | 1□ | 2□ | 3□ | 4□ | 5□ |
| 6.When it’s time for exercise, I can’t help but want to exercise | 1□ | 2□ | 3□ | 4□ | 5□ |
| 7. I like everything related to sports | 1□ | 2□ | 3□ | 4□ | 5□ |
| 8.No matter how busy I am, I can always find time to exercise | 1□ | 2□ | 3□ | 4□ | 5□ |

**Supplementary file 2：**

Please read each item carefully to understand its meaning, and then choose the answer that best suits you according to your actual situation, and tick the corresponding number with "√".

(1.Totally inconsistent;2.Not very consistent;3.Some match;4.More in line with;5.Completely suitable )

| **The exercise habits scale** | | | | | |
| --- | --- | --- | --- | --- | --- |
| 1.I have a habit of exercising | 1□ | 2□ | 3□ | 4£ | 5£ |
| 2. I always exercise consciously | 1□ | 2□ | 3□ | 4□ | 5□ |
| 3. Exercise is indispensable in my life | 1□ | 2□ | 3□ | 4□ | 5□ |
| 4. During the exercise, no matter what movement I do, I can do it easily. | 1□ | 2□ | 3□ | 4□ | 5□ |
| 5. I'm not used to living without exercise | 1□ | 2□ | 3□ | 4□ | 5□ |
| 6. Exercise is a hobby of mine | 1□ | 2□ | 3□ | 4□ | 5□ |
| 7. I like to participate in exercise activities | 1□ | 2□ | 3□ | 4□ | 5□ |
| 8. In my free time, I participate in exercise as much as possible | 1□ | 2□ | 3□ | 4□ | 5□ |
| 9. I think it’s easy to exercise | 1□ | 2□ | 3□ | 4□ | 5□ |
| 10. I feel very excited when I say exercise | 1□ | 2□ | 3□ | 4□ | 5□ |

**Supplementary file 3：**

Please read each item carefully to understand the meaning, and then according to your actual feelings in the last week, choose the answer that suits you best and tick the corresponding number with "√".

(1.Totally inconsistent;2.Not very consistent;3.Some match;4.More in line with;5.Completely suitable )

| **The Physical Health Belief Questionnaire** | | | | | |
| --- | --- | --- | --- | --- | --- |
| 1. If you don’t exercise, your health will get worse | 1□ | 2□ | 3□ | 4£ | 5£ |
| 2. When I am in poor physical health, I feel a sense of urgency to exercise | 1□ | 2□ | 3□ | 4□ | 5□ |
| 3.Poor physical health is prone to many diseases | 1□ | 2□ | 3□ | 4□ | 5□ |
| 4. I have a fear of being sick | 1□ | 2□ | 3□ | 4□ | 5□ |
| 5. Physical exercise can enhance physical health | 1□ | 2□ | 3□ | 4□ | 5□ |
| 6. Every time I get sick, I feel scared | 1□ | 2□ | 3□ | 4□ | 5□ |
| 7. Lack of exercise may cause some serious illness | 1□ | 2□ | 3□ | 4□ | 5□ |
| 8. Physical exercise can prevent diseases and prolong life | 1□ | 2□ | 3□ | 4□ | 5□ |
| 9. I felt a serious threat to my physical health when I was sick | 1□ | 2□ | 3□ | 4□ | 5□ |
| 10. When I am in poor physical health, I will overcome difficulties and exercise | 1□ | 2□ | 3□ | 4□ | 5□ |
| 11. Physical fitness evaluation is an important way for me to understand health | 1□ | 2□ | 3□ | 4□ | 5□ |
| 12. Physical exercise can delight the body and mind | 1□ | 2□ | 3□ | 4□ | 5□ |
| 13. Poor physical fitness will seriously affect my quality of life | 1□ | 2□ | 3□ | 4□ | 5□ |
| 14.When I am in poor health, I can overcome obstacles such as weather and environment to exercise | 1□ | 2□ | 3□ | 4□ | 5□ |
